# Complementary nutritional and health promoting constituents in germinated and probiotic fermented flours from cowpea, sorghum and orange fleshed sweet potato

**DOI:** 10.1038/s41598-024-52149-6

**Published:** 2024-01-23

**Authors:** Yusuf Olamide Kewuyemi, Oluwafemi Ayodeji Adebo

**Affiliations:** Food Innovation Research Group, Department of Biotechnology and Food Technology, Faculty of Science, University of Johannesburg, Doornfontein, P.O. Box 17011, Johannesburg, 2028 Gauteng South Africa

**Keywords:** Biotechnology, Applied microbiology

## Abstract

Germination and fermentation are age-long food processes that beneficially improve food composition. Biological modulation by germination and probiotic fermentation of cowpea, sorghum, and orange-fleshed sweet potato (OFSP) and subsequent effects on the physicochemical (pH and total titratable acidity), nutritional, antinutritional factors and health-promoting constituents/properties (insoluble dietary fibres, total flavonoid and phenolic contents (TFC and TPC) and antioxidant capacity) of the derived flours were investigated in this study. The quantification of targeted compounds (organic acids and phenolic compounds) on an ultra-high performance liquid chromatography (UHPLC) system was also done. The whole cowpea and sorghum were germinated at 35 °C for 48 h. On the other hand, the milled whole grains and beans and OFSP were fermented using probiotic mesophilic culture at 35 °C for 48 h. Among the resultant bioprocessed flours, fermented sorghum and sweet potato (FSF and FSP) showed mild acidity, increased TPC, and improved ferric ion-reducing antioxidant power. While FSF had better slowly digestible and resistant starches and the lowest oxalate content, FSP indicated better hemicellulose, lowest fat, highest luteolin, caffeic and vanillic acids. Germinated cowpea flour exhibited reduced tannin, better lactic acid, the highest crude fibre, cellulose, lignin, protein, fumaric, l-ascorbic, trans-ferulic and sinapic acids. The comparable and complementary variations suggest the considerable influence of the substrate types, followed by the specific processing-based hydrolysis and biochemical transitions. Thus, compositing the bioprocessed flours based on the unique constituent features for developing functional products from climate-smart edibles may partly be the driver to ameliorating linked risk factors of cardiometabolic diseases.

## Introduction

Africa’s triple burden of malnutrition (undernutrition, macro and micro-nutrient deficiency, as well as overnutrition) is an escalating human problem, and the detrimental impact of climate change exacerbates the situation^1,2^. Particularly, the continued rising prevalence and adverse outcomes like cardiometabolic diseases driven by linked risk factors are anticipated to severely affect the health, social and economic status of not just low but also middle and high-income countries^3^. The sustainable shift to better food utilisation of climate-resilient crops rich in essential nutrients and biologically active components is thus crucial for addressing malnutrition as well as preventing and managing dietary-related health issues^4,5^. Non-conventional but climate-smart food sources indigenous to sub-Saharan Africa include cowpea (*Vigna unguiculata*), sorghum (*Sorghum bicolor*) and OFSP (*Ipomoea batatas*)^5,6^. These edible substrates comprise basic nutrients (carbohydrates, minerals, protein, fibre, vitamins, etc.) and diverse functional fractions like phenolic compounds (e.g., phenolic acids and flavonoids), OAs, nondigestible dietary fibres, slowly digestible and resistant starches^5–9^). Noort et al.^5^ noted that systematic food processing strategies are required to explore the fortification potentials of the valuable components in the edibles and convert them into attractive, convenient, and health-enhancing foods.

Food components such as RS have related physiological effects to dietary fibres; thus, both can function as good prebiotic supplies for the proliferation of gut microflora to produce short-chain fatty acids for improved colonic health^10–12^. The starch is also involved in modulating postprandial blood glucose and attenuating blood-cholesterol levels^10,12^. The intake of SDS delivers slow and prolonged release of blood glucose, which may potentially aid in reducing insulin resistance and blood lipid levels, stabilise glucose metabolism, promote satiety and extend physical performance for weight control^13^. Insoluble dietary fibres such as cellulose and lignin have a good swelling capacity, which can limit food intake and exert a lubricating effect to aid intestinal motility and peristalsis^11^. Specifically, the cellulose chains possess a stable fibrous network that allows them to exhibit oil holding, cholesterol and glucose adsorption capacities, such that the fibre can absorb fat, sugar, and cholesterol, resulting in less contact with the human intestinal tract^11^. Beyond the antioxidant potency of phenolic compounds that can protect the human body from free radical harm, their role in anti-cardiometabolic diseases has equally been documented^14,15^. In a similar role, OAs are involved in the metabolism regulation of the human systems and can be an energy source^9^.

The highlighted and other reported physiological benefits of these key constituents, their presence, and desirable interactions during the processing of edible materials are indeed of huge interest to ameliorate the risk factors of cancers, diabetes, obesity, intestinal diseases and other cardiometabolic diseases. Lactic acid fermentation and germination are simple bioprocessing approaches that enrich numerous vital nutrients, biologically active compounds and phytocomponents with reductions in ANFs. These desirable modifications in the complex processes are achieved through various mechanisms enabled by microbial activity, activated and synthesised hydrolytic enzymes (viz. amylases, proteases, xylanases)^16,17^. Typical intermediate products from lactic acid fermentation or germination of grains are sourdough and germinated grains, which can be converted into flour. Despite the interesting potentials of the flours, the germinated counterparts are less suitable for subsequent conventional food processing into finished products because of excessive hydrolysing enzyme actions (e.g., dough-making process)^18^.

Attempts to partially substitute wheat flour with germinated ayocote bean flour^19^, germinated wheat or spelt flours^20,21^ at ≤ 30% improved the nutritional characteristics and phenolic compounds of the resultant flour, dough, and bread, but low replacement levels (5% or 10%) were suggested to be practicable for desirable physical and sensory features. Extended short-term germination, up to 72 h, negatively impacted bread quality^20^. Perri et al.^22^ exploited a sourdough fermentation approach, where germinated barley flour or grain (10%) and wheat flour (90%) were fermented and used at 20% to prepare sourdough bread. The authors reported that fermentation of the germinated flour or grain considerably lessened the negative textural problems (almost sticky, lowest cohesiveness and hardness) impacted by invasive enzymes in sprouted barley bread. The sourdough bread containing 10% of the germinated flour or grain showed improved nutritional contents and was sensorially and structurally preferred like the control wheat bread^22^.

Furthermore, the authors achieved compromises between the nutritional and technological features at low levels of incorporating germinated flours in bread preparation. Still, the content of the global nutritional and functional components is less than in the whole bioprocessed flours or end products. In an attempt to explore the applicability of the bioprocessed flour, spontaneously fermented cowpea and germinated quinoa flours may be composited at higher ratios for complementary and enhanced nutritional, functional and structural attributes in three-dimensional printed biscuits^23,24^. Expanding the range of germinated substrates for systematic use with fermented ones could facilitate a balance of nutritional, health-promoting components and desirable technological characteristics.

According to Gänzle and Gobbetti^25^, sourdough microorganisms are mainly LAB, and their actions lead to various important modifications in the derived products. In the same vein, germinated flours from cereals (barley and wheat), a pseudocereal (quinoa) and legumes (chickpea and lentil) have also been shown to be dominant of LAB strains with diverse microbiota^26^. Since microbial activities are modified during germination, this present work explored the same temperature (35 °C) suitable for the growth of probiotic mesophilic LAB culture during fermentation for germination of whole grain cereal (sorghum) and legume pulse (cowpea). A tuberous root vegetable (OFSP) was additionally fermented, all for a short-term duration (48 h). This is a controlled fermentation approach with more bioprocessed substrates and evaluated components compared to a prior study that investigated spontaneously fermented and germinated cowpea and quinoa at 28 °C for 48 h^27^. Moreover, this study hypothesised that the microbial metabolic activities during both bioprocesses would result in complementary physicochemical, nutritional, targeted phenolic compounds and OAs.

## Materials and methods

### Materials and edible material pre-processing

Whole cowpea, whole sorghum, and OFSP were sourced from the Agricultural Research Council, Nelspruit, Fish on Tackle Store, Kempton Park, and Woolworths store, Johannesburg, respectively, all in South Africa. The required chemicals and reagents (analytical grades) were procured from Merck (Darmstadt, Germany) and other notable manufacturers. The WGB and OFSP were dry-cleaned to remove foreign materials. The grains were divided into two batches, where batch A was subjected to the germination process, and batch B was milled in Philips Mill HR2056/90 (Koninklijke Philips N.V., Eindhoven, Netherlands) to prepare fermented flours. The OFSP was thinly peeled, rinsed with water, diced into cubes, lyophilised (Telstar LyoQuest freeze dryer, Terrassa, Spain) and then milled to obtain flour for fermentation. The flours were packed in ziplock bags and stored at 4 °C before further processing.

### Raw and bioprocessing of WGB and OFSP into flours

A portion of the raw flours described in “Materials and edible material pre-processing” was passed through a 500 µm mesh sieve (Analysette 3 Spartan, Fritsch GmbH, Idar-Oberstein, Germany) for quality comparisons with the bioprocessed flours. The bioprocessing techniques employed were probiotic fermentation and germination. For the controlled fermentation, 0.40 g (manufacturer recommendation) of freeze-dried culture (comprising *Lactococcus lactis* subsp. *lactis* biovar. *diacetylactis*, *Lactococcus lactis* subsp. *cremoris*, *Lactococcus lactis* subsp. *lactis*, *Leuconostoc mesenteroides* and *Leuconostoc pseudomesenteroides*, CHN-22, Chr. Hansen Holding A/S, Hørsholm, Denmark) was mixed with 100 g flour and 100 mL sterilised distilled water and then fermented at 35 °C for 48 h. On the other hand, the cleaned WGB were sterilised in sodium hypochlorite solution (0.5%, v/v) for 15 min and thoroughly rinsed several times with fresh distilled water^28^. The drained WGB were soaked in water (1:1, w/v) at 35 °C for 6 h and germinated with intermittent moistening for 48 h at 35 °C. The derived fermented doughs and germinated WGB with elongated radicles (germination sensu stricto) were lyophilised using a bench-top LyoQuest freeze dryer (Telstar, Spain) for 24 h at − 55 °C. The freeze-dried materials were milled (Philips, Netherlands) and passed through a 500 µm mesh sieve (Fritsch, Germany) to obtain bioprocessed flours.

### pH and TTA

20 mL of the recovered supernatant from a 10% flour suspension in distilled water was used to determine the pH and TTA. The pH was recorded using an Eutech Pte pH meter (pH 510, Taus, Singapore). For the TTA, the supernatant was titrated with sodium hydroxide solution (NaOH, 0.1 N) to pH 8.3^27^. The TTA of the flour samples were presented as mL of NaOH (0.1 N).

### Targeted OAs

The procedure reported by Tang et al.^29^ was slightly modified to determine the targeted OAs in the extract of the prepared flours. In brief, the extract was obtained by mixing 1 g flour with 15 mL 0.02 mol/L NaH_2_PO_4_ (pH 2.7) and then centrifuging (10 min, 6800×*g*, Eppendorf AG 5452, Hamburg, Germany) the mix to recover the sample supernatant. After that, the supernatant was diluted with 0.02 mol/L NaH_2_PO_4_ (pH 2.7) (1:3, v/v) and filtered into amber vials for analysis on a UHPLC-PDA (Shimadzu Corporation, Kyoto, Japan). The extract (10 µL) was injected and eluted through a Shim-pack GIST column C18 (2.1 × 100 mm, 3 µm particle size) (Shimadzu, Japan) with an isocratic mobile phase (0.02 mol/L NaH_2_PO_4_ (pH 2.7) at 0.25 mL/min flow rate within the column oven set at 30 °C. The acquisition time was 13 min per run. The reference standard (formic, fumaric, l-ascorbic, lactic, maleic, oxalic, propionic, shikimic, and succinic acids) calibration curves were used to calculate the concentrations (mg/kg) of the OAs in the sample extracts.

### TFC, TPC, targeted phenolic compounds and antioxidant capacities

#### Methanolic flour extraction

The extraction was done by mixing the flour (ca. 0.25 g) with 80% aqueous methanol containing 1% (HCl), and the mix was subsequently vortexed (K-550-GE, Scientific Industries, Inc., NY., USA) and sonicated using a water bath ultrasonicator (Argolab AU-220, Carpi, Italy) for 1 h. The recovered extract tubes were centrifuged (10 min, 4 °C, 2900×*g*, Eppendorf 5702R, Hamburg, Germany) to obtain supernatant and then filtered^24^ for subsequent analyses.

#### TFC and TPC

The TFC of the flour extracts were assayed following the modified protocol by Moyo et al.^30^. The reaction mixture (2.5% NaNO_2_, 1.25% AlCl_3_, 2% NaOH and 10 µL extract) and quercetin concentrations (standard) were separately contained in a 96-well plate and then read at 450 nm using a microplate smartReader™ 96 (MR-9600, Accuris Instruments, Benchmark Scientific Inc., NJ, USA). The TFC was recorded as the quercetin (mg) equivalent per g sample. The TPC was determined with slight changes based on the Folin-Ciocalteu reagent procedure^31^. The reaction mixture [diluted Folin-Ciocalteu phenol reagent (50 µL), 7.5% Na_2_CO_3_ and 10 µL extract] and gallic acid standard concentrations were separately contained in a 96-well plate. The plate was then incubated for 30 min and read at 750 nm using a microplate smartReader™ 96 (Accuris Instruments, USA). The TPC was calculated as gallic acid (mg) equivalent per g sample.

#### Targeted phenolic compounds

A UHPLC-PDA (Shimadzu, Japan) was used to determine and quantify the targeted phenolic compounds (flavonoids: luteolin, taxifolin, and quercetin; phenolic acids: caffeic, trans-ferulic, gallic, *p*-coumaric, sinapic, and vanillic) based on the standard calibration curves (µg/g). An earlier detailed operating condition, column type and solvent [Phase A: Milli-Q water and 0.10% formic acid, Phase B: acetonitrile (49.95%), methanol (49.95%), and formic acid (0.10%)] gradient program was used^24^.

#### Antioxidant capacities

The ABTS free radical scavenging activity of the prepared extracts was experimented with using the Sadh et al.^32^ protocol. The mixed solution of the extract and the ABTS working solution in a 96-well plate was incubated (30 min, at room temperature), and the absorbance of the reacted mixture and the blank were read at 734 nm on a microplate smartReader™ 96 (Accuris Instruments, USA). The percentage inhibition of the ABTS radical was calculated using the recorded absorbances. For the FRAP assay, the required solutions, including acetate buffer (300 mM, pH 3.6), diluted HCl (40 mM), 2,4,6-Tripyridyl-S-triazine (TPTZ, 10 mM) and ferric chloride hexahydrate (FeCl_3_⋅6H_2_O, 20 mM) were prepared fresh for the assay^33^. Two hundred and forty microliters (240 µL) of the FRAP working solution (at 37 °C) were pipetted into each plate well, followed by 10 µL of the standard (Trolox solution, 0 to 1 mM), 10 µL of the methanolic extracts to another series of wells, and the blank control was 75% ethanol^34^. Next, the plate was incubated at 37 °C for 30 min (Model: 222/227, Scientific Manufacturing CC, Cape Town, South Africa), and the derived reaction mixture absorbance was read at 593 nm on a microplate smartReader™ 96 (Accuris Instruments, USA). The FRAP of the extracts were further presented as a millimolar of Trolox equivalent per gram (mM TE/g).

#### Tannin and oxalate contents

The tannin content in the flours was analysed following the reported methods by Price^35^ and Adebiyi et al.^36^, with slight alterations. The flour samples were extracted by adding 25 mL of 1% HCl in methanol to 500 mg flour and held at 30 °C for 20 min (Labcon, Krugersdorp, South Africa). The mixture was sonicated for 2 h using a water bath ultrasonicator (Argolab, Italy) and then centrifuged for 10 min at 2100×*g* (Eppendorf, Germany). The vanillin reagent (5 mL) was added to 1 mL of the resulting supernatant (sample extract). On the other hand, 5 mL of concentrated HCl (4%) in methanol was pipetted into a 15 mL tube containing 1 mL of the corresponding sample extract (blank) and vortexed (Scientific Industries, Inc., USA). The mixtures were pipetted into a 96-well plate and held for 20 min at 30 °C (Scientific Manufacturing CC, South Africa). A microplate smartReader™ 96 (Accuris Instruments, USA) was used to record the reaction mixture absorbance at 500 nm. The differences in the absorbances were calculated, and the tannic acid standard curve was used to determine the tannin content in the extracts as mg tannic acid equivalent per g. The oxalate content determination involved the addition of 75 mL of 3 M H_2_SO_4_ into a 100 mL conical flask containing 1 g of flour. The mixture was intermittently stirred magnetically for 1 h and filtered through a filter paper (Whatman no. 1). The filtrate (20 mL) was afterwards titrated against hot (80 to 90 °C) potassium permanganate solution (0.1 N) until the appearance of a faint pink colour (at least for 30 s). The oxalate concentrations were calculated based on 1 mL of 0.1 N potassium permanganate equals 0.006303 g oxalate^36^.

### Proximate composition and insoluble dietary fibres

The proximate components of the flour samples were analysed according to the AOAC^37^ standard methods. The ash (923.03, 32.1.05), crude fibre (Ba 6a-05), fat (920.39-A), and protein (990.03, 6.25 as the nitrogen conversion factor) contents. Total carbohydrate content was calculated based on the difference between 100 and other proximate composition parameters. The general Atwater factor was used to express the total energy of the samples^38^. The acid and neutral detergent fibres (ADF and NDF) were determined according to the method by Van Soest et al.^39^ using the filter bag techniques for the Ankom A200/A200l fibre analyser (Ankom Technology, New York, USA). The ADF was determined as the residue left after digesting ca. 500 mg of the flour sample with H_2_SO_4_ and cetyltrimethylammonium bromide. On the other hand, the NDF was determined as the residue left after digesting ca. 500 mg of the flour sample with neutral detergent, heat-stable alpha-amylase and Na_2_SO_3_ solutions. The difference between the calculated ADF and NDF was recorded as the HMC content.

### In vitro starch digestibility

The slightly modified in vitro starch digestibility procedure reported by Kaur and Sandhu^40^ was adopted for the digestibility assay. Briefly, 1 mL amyloglucosidase was suspended in a test tube containing deionised water (2 mL). In a 50 mL centrifuge tube containing distilled water (25.7 mL), porcine pancreatic alpha-amylase (3.89 g) was dispersed and centrifuged (2500×*g*, 10 min, Eppendorf, Germany). The obtained supernatant (18.7 mL) was mixed with the diluted amyloglucosidase (1 mL) to prepare a fresh enzyme solution. 10 mL of guar gum (5 g/L) and 5 mL of sodium acetate (0.5 M) aliquots were transferred to a 200 mL scotch bottle containing 0.5 g of flour. To this mixture, 5 mL of enzyme solution and 15 glass balls (5 mm) were added and subsequently incubated in a shaking ecobath water bath (170 rpm, 37 °C) (Labotec, Midrand, South Africa). The aliquot (0.5 mL) pipetted periodically was mixed with ethanol (80%, 4 mL), and the glucose concentrations in the reaction mixture were measured with glucose oxidase and peroxidase assay kits. The starch classification based on digestibility was: (a) RDS (hydrolysed within 20 min of incubation), (b) SDS (digested starch between 20 and 120 min of incubation), and (c) RS (unhydrolysed starch after 120 min).

### Statistical analysis

The experimental data generated in replicate was statistically evaluated based on a one-way analysis of variance (IBM SPSS software, ver. 26.0, NY, USA). The computed averages were compared using the Tukey test (p ≤ 0.05 significance probability level) and presented as the average ± standard deviation. On the same software, Pearson’s correlation was done using a two-tailed test of significance at p ≤ 0.05 probability level.

### Ethical statement

The authors confirmed that the use of the investigated plant-based materials (cowpea, sorghum, and orange-fleshed sweet potato) in our study complied with the relevant institutional, national, and international guidelines and legislation, in particular, the IUCN Policy Statement on Research Involving Species at Risk of Extinction.

## Results and discussion

### pH, TTA and targeted OAs

**Figure 1:**
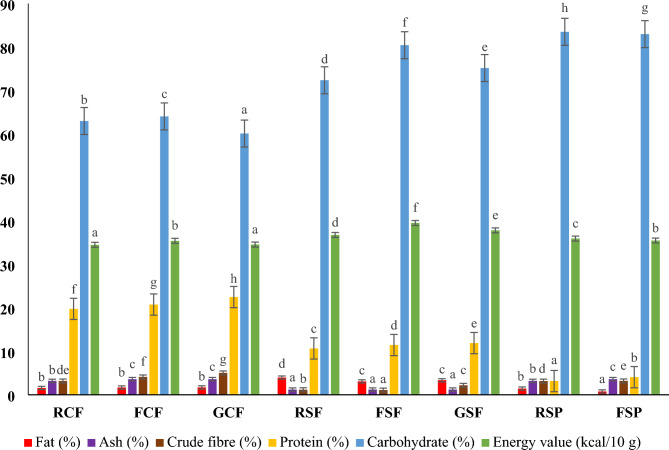
Proximate composition of the raw, fermented, and germinated WGB and OFSP flours. Means shown as bars with varying superscripts are significantly different (p ≤ 0.05). *RCF* raw cowpea flour, *RSF* raw sorghum flour, *RSP* raw, orange-fleshed sweet potato flour, *FCF* fermented cowpea flour, *FSF* fermented sorghum flour, *FSP* fermented orange-fleshed sweet potato flour, *GCF* germinated cowpea flour, *GSF* germinated sorghum flour, *WGB* whole grains and beans, *OFSP* orange-fleshed sweet potato flour.

The biochemical properties, including pH and TTA of the raw, fermented, and germinated flours at the same temperature and time, are presented in Table 1. The significantly (p ≤ 0.05) high pH in the RCF (6.57), RSF (6.49) and RSP (6.18) reduced to lower levels in the derived GCF (6.20), GSF (5.82), FSP (4.52), FCF (4.49), and FSF (3.89). Accordingly, the reduced pH values in the flours coincided with increases in the TTA levels of the bioprocessed flours (GSF, 1.38 mL/g), (GCF, 2.26 mL/g), (FSF, 2.46 mL/g), (FSP, 3.00 mL/g), and (FCF, 4.25 mL/g), Table 1. Unsurprisingly, Pearson’s correlation showed a negative and significant (p ≤ 0.05) relationship (R^2^ =  − 0.79) between the pH and TTA (Table 2). This implies an inverse trend of decreased pH would result in high TTA and vice versa. The notable high acidity in FCF may be ascribed to the hydrolysed carbohydrate in the presence of a high nitrogen source (protein, Fig. 1) into elevated levels of simple monomers. These sources enhanced microbial activity and the subsequent formation of OAs (Table 3).

**Table 1:** pH, TTA, TFC, TPC, ANFs and antioxidant activities of the raw, fermented, and germinated WGB and OFSP flours.

Samples	pH	TTA (mL/g)	TFC (mg QE/g)	TPC (mg GAE/g)	Tannin content (mg TAE/g)	Oxalate content (mg/g)	ABTS (% inhibition)	FRAP (mM TE/g)
RCF	6.57^g^ [0.03]	0.85^b^ [0.02]	11.82^e^ [0.15]	1.61^g^ [0.02]	7.79^d^ [0]	3.47^ef^ [0]	67.46^g^ [0.24]	13.56^f^ [0.21]
FCF	4.49^b^ [0.01]	4.25^g^ [0.12]	14.16^f^ [0.22]	1.91^h^ [0.02]	6.02^c^ [0]	2.52^d^ [0]	78.75^h^ [0.63]	15.98^g^ [0.43]
GCF	6.20^e^ [0.00]	2.26^d^ [0.06]	2.98^a^ [0.03]	0.83^f^ [0.01]	4.89^b^ [0]	1.73^c^ [0]	40.46^f^ [0.21]	8.42^e^ [0.17]
RSF	6.49^f^ [0.01]	0.14^a^ [0.01]	3.75^c^ [0.10]	0.17^a^ [0.01]	6.00^c^ [0]	0.63^ab^ [0]	6.59^a^ [0.00]	2.12^a^ [0.04]
FSF	3.89^a^ [0.01]	2.46^e^ [0.02]	4.12^d^ [0.02]	0.36^b^ [0.00]	5.84^c^ [0]	0.44^a^ [0]	11.22^c^ [0.42]	3.71^b^ [0]
GSF	5.82^d^ [0.00]	1.38^c^ [0.02]	3.33^b^ [0.07]	0.48^c^ [0.02]	4.06^a^ [0]	0.85^b^ [0]	9.75^b^ [0.21]	4.78^cd^ [0.21]
RSP	6.18^e^ [0.00]	0.92^b^ [0.03]	3.84^cd^ [0.06]	0.58^d^ [0.01]	22.90^f^ [0]	3.15^e^ [0]	19.64^e^ [0.00]	4.51^c^ [0.02]
FSP	4.52^c^ [0.00]	3.00^f^ [0.11]	2.85^a^ [0.03]	0.71^e^ [0.01]	16.42^e^ [0]	2.21^cd^ [0]	18.37^d^ [0.00]	5.35^d^ [0.21]

Values in square brackets are the standard deviations of the respective means with varying superscripts that are significantly different (p ≤ 0.05) per column.

*TTA* total titratable acidity, *TFC* total flavonoid content, *TPC* total phenolic content, *ABTS* 2,2-azinobis-(3-ethylbenzthiazolin-6-sulfonic acid), *FRAP* ferric ion reducing antioxidant power, *WGB* whole grains and beans, *OFSP* orange-fleshed sweet potato, *RCF* raw cowpea flour, *RSF* raw sorghum flour, *RSP* raw, orange-fleshed sweet potato flour, *FCF* fermented cowpea flour, *FSF* fermented sorghum flour, *FSP* fermented orange-fleshed sweet potato flour, *GCF* germinated cowpea flour, *GSF* germinated sorghum flour.

**Table 2:** Pearson’s correlation between the pH, TTA and targeted organic acids in the extracts from raw and bioprocessed flours.

	pH	TTA	Formic	Fumaric	Lactic	l-Ascorbic	Maleic	Oxalic	Propionic	Shikimic	Succinic
pH	1	− 0.79*	0.10	0.39	− 0.43	0.50	− 0.68	− 0.19	− 0.32	− 0.22	− 0.41
TTA	− 0.79*	1	0.43	0.06	0.72*	− 0.16	0.83*	0.64	0.73*	0.65	0.71*
Formic	0.10	0.43	1	0.86	0.30	0.57	0.25	0.50	0.78*	0.76*	0.58
Fumaric	0.39	0.06	0.86	1	0.01	0.89	− 0.21	0.36	0.36	0.33	0.09
Lactic	− 0.43	0.72*	0.30	0.01	1	− 0.13	0.43	0.88	0.61	0.53	0.47
l-Ascorbic	0.50	− 0.16	0.57	0.89	− 0.13	1	− 0.49	0.30	− 0.02	− 0.03	− 0.31
Maleic	− 0.68	0.83*	0.25	− 0.21	0.43	− 0.49	1	0.26	0.68	0.67	0.84
Oxalic	− 0.19	0.64	0.50	0.36	0.88	0.30	0.26	1	0.52	0.46	0.30
Propionic	− 0.32	0.73*	0.78*	0.36	0.61	− 0.02	0.68	0.52	1	0.97	0.93
Shikimic	− 0.22	0.65	0.76*	0.33	0.53	− 0.03	0.67	0.46	0.97	1	0.94
Succinic	− 0.41	0.71*	0.58	0.09	0.47	− 0.31	0.84	0.30	0.93	0.94	1

*TTA* total titratable acidity.

*The observed correlation is significant at the p ≤ 0.05 level.

**Table 3:** Targeted organic acids (mg/kg) in the extracts from raw, fermented, and germinated WGB and OFSP flours.

Samples	Formic acid λ 205 nm	Fumaric acid λ 208 nm	Lactic acid λ 203 nm	l-Ascorbic acid λ 213 nm	Maleic acid λ 210 nm	Oxalic acid λ 190 nm	Propionic acid λ 206 nm	Shikimic acid λ 190 nm	Succinic acid λ 190 nm
Rt (min)	1.27	2.20	1.67	1.51	1.97	1.05	4.21	7.68	8.37
RCF	528.13^a^ [6.51]	3698.12^c^ [65.56]	ND	1895.04^d^ [23.43]	ND	6271.85^c^ [51.87]	7.71^b^ [0.64]	23.82^ab^ [2.30]	23.41^a^ [0.77]
FCF	685.52^c^ [1.90]	1563.89^a^6.43]	251.44^d^ [2.07]	ND	1802.99^f^ [5.42]	18,905.15^f^ [3.89]	67.83^d^ [0.84]	86.37^d^ [0.07]	100.95^b^ [0.95]
GCF	569.68^b^ [11.04]	3435.03^b^ [1.77]	234.46^d^ [0.64]	2330.11^e^ [4.67]	ND	25,839.58^h^ [83.30]	24.29^c^ [3.87]	42.01^c^ [3.63]	25.82^a^ [1.05]
RSF	ND	ND	46.90^a^ [0.40]	195.79^a^ [15.10]	38.92^ab^ [10.40]	3224.75^a^ [22.47]	ND	29.46^b^ [5.05]	27.97^a^ [3.10]
FSF	ND	ND	127.69^b^ [18.22]	155.55^a^ [2.03]	442.44^c^ [1.66]	6749.45^d^ [92.39]	7.88^b^ [0.30]	18.40^a^ [0]	25.82^a^ [0.06]
GSF	ND	ND	45.42^a^ [7.73]	473.42^c^ [8.68]	754.89^d^ [28.98]	4952.00^b^ [140.43]	ND	19.80^a^ [0.24]	28.02^a^ [0.88]
RSP	ND	ND	197.12^c^ [0.18]	ND	62.20^b^ [0.86]	13,855.64^e^ [28.64]	5.40^ab^ [0.11]	17.25^a^ [0.11]	23.14^a^ [0.01]
FSP	ND	ND	187.65^c^ [0.59]	287.82^b^ [0.95]	909.92^e^ [3.61]	19,210.34^g^ [33.06]	0.50^a^ [0.01]	19.15^a^ [1.20]	26.28^a^ [2.17]

Values in square brackets are the standard deviations of the respective means with varying superscripts that are significantly different (p ≤ 0.05) per column.

*WGB* whole grains and beans, *OFSP* orange-fleshed sweet potato, *ND* not detected, *Rt* retention time, *RCF* raw cowpea flour, *RSF* raw sorghum flour, *RSP* raw orange-fleshed sweet potato flour, *FCF* fermented cowpea flour, *FSF* fermented sorghum flour, *FSP* fermented orange-fleshed sweet potato flour, *GCF* germinated cowpea flour, *GSF* germinated sorghum flour.

As reflected in Table 1, the acidity levels were more pronounced in fermented than germinated flours, probably due to the differential metabolic activity during the processes^17,26^. As in the case of the fermented flours, the action of the mixed culture comprising homofermentative and heterofermentative LAB to break down carbohydrates resulted in the build-up of OAs. Consequently, it reduced the pH and increased the TTA levels^41–43^. In contrast, carbohydrates present in the germinating grains (Fig. 1) might have been partly used to synthesise other reserve grain constituents^44^ or the plethora of microbes present during germination was less effective in lowering the pH of the GCF and GSF as compared to the LAB fermented flours^26,45^. Previous investigations have reported higher acidity in fermented flours than germinated flours from cowpea, pearl millet, and quinoa^27,45^.

OAs are naturally existing or chemically synthesised secondary metabolites in foods with activities related to the acidic pH and undissociated nature of the acids^46^. Beyond the reported potent anti-microbial activity of specific OAs (lactic and propionic acids) for food preservation^46^, OAs may be an energy source, impact the taste characteristics of food and be involved in the metabolism regulation of the human systems^9^. The elevated TTA contents and associated low pH values of the fermented or germinated flours due to the process-based microbial and enzymatic activities were consistent with the enhanced concentrations of the quantified OAs in the bioprocessed flours. Indeed, Pearson’s correlation in Table 2 revealed positive and significant coefficient correlations (p ≤ 0.05) between the TTA and some OAs such as lactic (R^2^ = 0.72), maleic (R^2^ = 0.83), propionic (R^2^ = 0.73), and succinic (R^2^ = 0.71).

Remarkably, the fermented flours, exhibiting high acidities (Table 1), had increased significant (p ≤ 0.05) levels of most OAs (Table 3). Amongst the dicarboxylic acids (fumaric, maleic, oxalic, and succinic), FCF had the highest concentrations of maleic (1802.99 mg/kg) and succinic (100.95 mg/kg) acids. During the fermentation metabolism (particularly glycolysis and the oxidative pentose-phosphate pathways), glucose is metabolised to phosphoenol pyruvate and further converted through the action of vital enzymes (fumarase, fumarate reductase, malate dehydrogenase, and phosphoenolpyruvate carboxykinase) to synthesise succinate^47^. Maleic acid is one of the accompanying derivatives in succinic acid production^47^, and such phenomena might have caused the observation in this study. The utilisation of succinic acid ranges from an acidulant, anti-microbial to flavouring agents^48^. Since the isomerisation of maleic acid results in the formation of fumaric acid, the undetected fumaric levels in FSF and FSP could imply that the available maleic acids in the flours were not sufficiently isomerised to form fumaric acid. Fumaric and succinic acids have been reported to influence a considerable masking effect on bitterness^9^. Notably, the reduced content of fumaric acid and high levels of total polyphenols (Table 1) in FCF may imply the bitter-tasting characteristic of the flour^9,49^.

The FCF repeatedly showed higher concentrations of the targeted monocarboxylic acids (formic: 685.52, propionic: 67.83, and shikimic: 86.37 mg/kg). Formate is derived from glycolysis via pyruvate formate lyase activity^50^. While shikimic acid is produced through the shikimate pathway by shikimate dehydrogenase conversion^51^, propionate, on the other hand, is generated via pathways that comprise 1,2-propanediol (intermediate), the acryloyl-CoA and the methylmalonyl-CoA or succinate pathways^52^. The positive Pearson’s correlations (p ≤ 0.05) recorded between formic and propionic (R^2^ = 0.78) and shikimic (R^2^ = 0.76) acids (Table 2) could be ascribed to a relationship in their metabolic pathways. The concentration of propionic acid was found to be significantly (p ≤ 0.05) correlated with gallic acid (R^2^ = 0.74) and *p*-Coumaric (R^2^ = 0.75) acids (Tables 4 and 5). This trend could be attributed to a plausible relationship between microbiota formation of propionate and an enhancement of this formation in the presence of phenolic acids. Jia et al.^53^ have also reported a similar trend. High percentages of formic and propionic acids were part of mixed OAs in a basal diet of corn-soybean meal that enhanced broiler chickens’ immune function and antioxidative properties^54^. Further suggested beneficial effects of propionic acid in the gut include elevated insulin sensitivity and lower cholesterol and triglyceride levels^55^.

**Table 4:** Pearson’s correlation between the targeted organic acids and phenolic compounds of the extracts from raw and bioprocessed flours.

	Formic	Fumaric	Lactic	l-Ascorbic	Maleic	Oxalic	Propionic	Shikimic	Succinic
Formic	1	0.86	0.30	0.57	0.25	0.50	0.78*	0.76*	0.58
Fumaric	0.86	1	0.01	0.89	− 0.21	0.36	0.36	0.33	0.09
Lactic	0.30	0.01	1	− 0.13	0.43	0.88	0.61	0.53	0.47
l-Ascorbic	0.57	0.89	− 0.13	1	− 0.49	0.30	− 0.02	− 0.03	− 0.31
Maleic	0.25	− 0.21	0.43	− 0.49	1	0.26	0.68	0.67	0.84
Oxalic	0.50	0.36	0.88	0.30	0.26	1	0.52	0.46	0.30
Propionic	0.78*	0.36	0.61	− 0.02	0.68	0.52	1	0.97	0.93
Shikimic	0.76*	0.33	0.53	− 0.03	0.67	0.46	0.97	1	0.94
Succinic	0.58	0.09	0.47	− 0.31	0.84	0.30	0.93	0.94	1
Caffeic	− 0.22	− 0.30	0.32	− 0.19	0.51	0.37	− 0.06	− 0.10	0.03
Trans-Ferulic	0.59	0.42	0.62	0.29	0.29	0.75*	0.59	0.64	0.45
Gallic	0.71	0.48	0.56	0.13	0.23	0.53	0.74*	0.65	0.58
Luteolin	− 0.69	− 0.70	0.15	− 0.43	0.13	0.09	− 0.43	− 0.35	− 0.27
*p-*Coumaric	0.68	0.43	0.72*	0.22	0.49	0.80*	0.75*	0.74*	0.60
Quercetin	− 0.85	− 0.85	0.03	− 0.60	− 0.19	− 0.24	− 0.50	− 0.45	− 0.36
Sinapic	− 0.38	− 0.20	0.32	0.16	− 0.33	0.40	− 0.40	− 0.38	− 0.52
Taxifolin	0.75*	0.34	0.54	− 0.05	0.75*	0.49	0.94	0.93	0.92
Vanillic	− 0.14	− 0.18	0.59	− 0.10	0.25	0.61	− 0.01	− 0.07	− 0.05

*The observed correlation is significant at the p ≤ 0.05 level.

**Table 5:** Pearson’s correlation between the targeted organic acids and phenolic compounds of the extracts from raw and bioprocessed flours.

	Caffeic	Trans-Ferulic	Gallic	Luteolin	*p*-Coumaric	Quercetin	Sinapic	Taxifolin	Vanillic
Formic	− 0.22	0.59	0.71	− 0.69	0.68	− 0.85	− 0.38	0.75*	− 0.14
Fumaric	− 0.30	0.42	0.48	− 0.70	0.43	− 0.85	− 0.20	0.34	− 0.18
Lactic	0.32	0.62	0.56	0.15	0.72*	0.03	0.32	0.54	0.59
l-Ascorbic	− 0.19	0.29	0.13	− 0.43	0.22	− 0.60	0.16	− 0.05	− 0.10
Maleic	0.51	0.29	0.23	0.13	0.49	− 0.19	− 0.33	0.75*	0.25
Oxalic	0.37	0.75*	0.53	0.09	0.80*	− 0.24	0.40	0.49	0.61
Propionic	− 0.06	0.59	0.74*	− 0.43	0.75*	− 0.50	− 0.40	0.94	− 0.01
Shikimic	− 0.10	0.64	0.65	− 0.35	0.74*	− 0.45	− 0.38	0.93	− 0.07
Succinic	0.03	0.45	0.58	− 0.27	0.60	− 0.36	− 0.52	0.92	− 0.05
Caffeic	1	0.19	− 0.32	0.65	0.29	0.04	0.40	0.12	0.79*
Trans-Ferulic	0.19	1	0.30	0.09	0.95	− 0.36	0.312	0.67	0.49
Gallic	− 0.32	0.30	1	− 0.64	0.43	− 0.45	− 0.45	0.60	− 0.14
Luteolin	0.65	0.09	− 0.64	1	− 0.03	0.68	0.71	− 0.31	0.61
*p-*Coumaric	0.29	0.95	0.43	− 0.03	1	− 0.47	0.17	0.82*	0.52
Quercetin	0.04	− 0.36	− 0.45	0.68	− 0.47	1	0.49	− 0.58	0.08
Sinapic	0.40	0.31	− 0.45	0.711	0.17	0.49	1	− 0.39	0.62
Taxifolin	0.12	0.67	0.60	− 0.31	0.82*	− 0.58	− 0.39	1	0.17
Vanillic	0.79*	0.49	− 0.14	0.61	0.52	0.08	0.62	0.17	1

*The observed correlation is significant at the p ≤ 0.05 level.

Higher l-ascorbic acid was detected in the GCF (2330.11 mg/kg) and GSF (473.42 mg/kg) compared to the fermented ones. A similar enhanced ascorbic acid content in germinated cowpea (20.44 mg/100 g) compared to the raw cowpea (8.18 mg/100 g) was previously shown (Khyade and Jagtap^56^). The sugar acid is a common antioxidant, and its biosynthesis during germination through the conversion of reducing sugars (d-glucose and l-galactose) could have had a role in modulating the emergence of the seed radicles^57,58^. Though l-ascorbic acid was not detected in FCF, a reduced concentration in FSF (155.55 mg/kg) and a small amount synthesised in the FSP (287.82 mg/kg) imply that the metabolised glucose during fermentation was less available to be converted to ascorbic acid.

The plausible increase in the calcium content of the bioprocessed flours^27^ might have been a response to the raised oxalic concentrations^59^, with the most striking increase recorded in GCF (25,839.58 mg/kg). Kanauchi et al.^59^ reported a high oxalic content (61%) in germinated barley rootlets compared to other measured components (acrospires and bran). Besides the probable radicle contributions to the oxalic acid levels, the even increase in the l-ascorbic acid of the germinated flours may have participated as an immediate precursor in the formation of oxalic acid^57^. Notwithstanding, the recorded oxalic acid increase in the fermented flours may partly be due to other source precursors like citrate, oxaloacetate and glyoxylate^59^. Sakouhi et al.^58^ reported that oxalic acid ameliorated the growth of a heavy metal-treated germinating chickpea and reversed the metal-induced oxidative stress. Although oxalic acid can be precipitated as calcium oxalate^59^, its moderate concentrations during germination sensu stricto could avert the detrimental effects of heavy metals.

The higher lactic acid (alpha-hydroxy) content in FCF (251.44 mg/kg) and FSF (127.69 mg/kg) than the raw counterparts conform with the literature as the microbial product of lactic acid fermentation and glucose metabolism. Similarly, GCF showed better lactic acid concentration (234.46 mg/kg) than GSF (45.42 mg/kg). According to Östman et al.^60^, the production/enrichment of lactic acid during sourdough fermentation can reduce human postprandial blood glucose and insulin responses.

### TFC, TPC, targeted phenolic compounds, and in vitro antioxidant capacities

The TFC, TPC, and antioxidant capacities of the extracts from raw and biomodified flours are shown in Table 1. The bioprocessed cowpea extracts had the highest TPC (0.83–1.91 mg GAE/g). In the same vein, the cowpea extracts exhibited the highest abundance of targeted phenolic acids, like gallic acid, *p*-coumaric acid, trans-ferulic acid, and a flavonoid (taxifolin), than other biomodified flours (Table 6). The findings aligned with the positive Pearson’s correlation (p ≤ 0.05) between TPC and gallic acid (R^2^ = 0.719) and taxifolin (R^2^ = 0.793) (Table 7). Correspondingly, gallic acid and taxifolin displayed significant (p ≤ 0.05) correlations with the antioxidant capacities expressed as percentage inhibition of ABTS (R^2^ = 0.759 and 0.788, respectively) and reduction of ferric iron (Fe^3+^) to ferrous iron (Fe^2+^) (R^2^ = 0.713 and 0.794, respectively) (Table 7). Thus, the high phenolic concentrations reported for the bioprocessed flour extracts (particularly for the fermented flours in Tables 1 and 6) seemingly influenced their resultant high antioxidant capacities (Table 1). The trends agree with earlier studies on the strong relationship between these antioxidant compounds and the antioxidant capacities of whole legume flour extracts^4,61^. In addition, WGB extracts of legumes (including cowpea beans) have been reported to exhibit higher phenolic acids and antioxidant capacity than assayed cereals (corn and wheat)^61^.

**Table 6:** Targeted phenolic compounds (µg/g) in the extracts from raw, fermented and germinated WGB and OFSP flours.

Samples	Caffeic acid λ 323 nm	Trans-Ferulic acid λ 322 nm	Gallic acid λ 271 nm	Luteolin λ 348 nm	*p*-Coumaric acid λ 309 nm	Quercetin λ 371 nm	Sinapic acid λ 319 nm	Taxifolin λ 290 nm	Vanillic acid λ 261 nm
Rt (min)	8.60	9.45	8.53	10.67	9.35	10.60	9.02	9.30	8.71
RCF	2.10^a^ [0.03]	794.34^b^ [69.77]	2908.31^c^ [11.49]	7.01^a^ [0.08]	708.80^c^ [57.40]	1.72^a^ [0.30]	0.55^a^ [0]	3771.00^d^ [138.87]	10,041.44^b^ [43.64]
FCF	5.99^d^ [0.06]	1253.44^de^ [15.09]	5630.93^f^ [5.91]	67.78^b^ [1.97]	1379.73^g^ [9.21]	48.18^b^ [1.72]	3.17^a^ [0.01]	10,651.61^g^ [3.41]	18,431.94^d^ [59.28]
GCF	5.04^c^ [0.04]	1340.56^e^ [2.15]	3266.43^d^ [2.20]	106.97^c^ [2.33]	1276.88^f^ [1.72]	76.39^c^ [1.24]	1448.32^e^ [50.79]	4229.58^f^ [10.20]	24,308.75^e^ [286.11]
RSF	ND	974.70^c^ [72.54]	97.00^a^ [1.93]	162.20^e^ [0.63]	688.78^c^ [44.29]	154.18^f^ [2.65]	822.11^c^ [58.26]	2319.27^b^ [84.42]	10,612.21^b^ [306.28]
FSF	6.91^e^ [0.19]	774.47^b^ [16.03]	227.12^ab^ [1.76]	126.36^d^ [9.32]	794.22^d^ [14.04]	121.51^e^ [1.51]	897.12^c^ [16.75]	3251.99^c^ [4.45]	25,226.70^f^ [12.26]
GSF	8.36^f^ [0.12]	523.06^a^ [23.50]	58.79^a^ [0.23]	150.43^e^ [8.27]	430.80^a^ [27.97]	128.95^e^ [3.74]	586.93^b^ [25.24]	1352.46^a^ [45.55]	9096.91^a^ [153.86]
RSP	2.63^b^ [0.15]	627.58^a^ [24.16]	4565.08^e^ [203.56]	105.19^c^ [12.88]	531.24^b^ [19.07]	153.80^f^ [2.88]	639.69^b^ [26.65]	1516.84^a^ [41.48]	17,614.48^c^ [555.18]
FSP	16.10^g^ [0.05]	1148.35^d^ [3.19]	324.63^b^ [5.57]	244.02^f^ [2.84]	1076.58^e^ [3.65]	109.14^d^ [4.55]	1281.98^d^ [5.49]	3982.65^e^ [18.14]	43,000.99^g^ [54.85]

Values in square brackets are the standard deviations of the respective means with varying superscripts that are significantly different (p ≤ 0.05) per column.

*WGB* whole grains and beans, *OFSP* orange-fleshed sweet potato, *ND* not detected, *Rt* retention time, *RCF* raw cowpea flour, *RSF* raw sorghum flour, *RSP* raw orange-fleshed sweet potato flour, *FCF* fermented cowpea flour, *FSF* fermented sorghum flour, *FSP* fermented orange-fleshed sweet potato flour, *GCF* germinated cowpea flour, *GSF* germinated sorghum flour.

**Table 7:** Pearson’s correlation between the TFC, TPC, targeted phenolic compounds and in vitro antioxidant activity of the extracts from raw and bioprocessed flours.

	TFC	TPC	Caffeic	Trans-Ferulic	Gallic	Luteolin	*p*-Coumaric	Quercetin	Sinapic	Taxifolin	Vanillic	ABTS	FRAP
TFC	1	0.92	− 0.25	0.20	0.62	− 0.73*	0.35	− 0.78*	− 0.85	0.76*	− 0.32	0.90	0.91
TPC	0.92	1	− 0.03	0.38	0.72*	− 0.66	0.54	− 0.88	− 0.66	0.79*	− 0.08	0.98	0.99
Caffeic	− 0.25	− 0.03	1	0.19	− 0.32	0.65	0.29	0.04	0.40	0.12	0.79*	− 0.17	− 0.08
Trans-Ferulic	0.20	0.38	0.19	1	0.30	0.09	0.95	− 0.36	0.31	0.67	0.49	0.44	0.40
Gallic	0.62	0.72*	− 0.32	0.30	1	− 0.64	0.43	− 0.45	− 0.45	0.60	− 0.14	0.76*	0.71*
Luteolin	− 0.73*	− 0.66	0.65	0.09	− 0.64	1	− 0.03	0.68	0.71	− 0.31	0.61	− 0.73*	− 0.70
*p*-Coumaric	0.35	0.54	0.29	0.95	0.43	− 0.03	1	− 0.47	0.17	0.82*	0.52	0.58	0.56
Quercetin	− 0.78*	− 0.88	0.04	− 0.36	− 0.45	0.68	− 0.47	1	0.49	− 0.58	0.08	− 0.90	− 0.91
Sinapic	− 0.85	− 0.66	0.40	0.31	− 0.45	0.71	0.17	0.49	1	− 0.39	0.62	− 0.61	− 0.63
Taxifolin	0.76*	0.79*	0.12	0.67	0.60	− 0.31	0.82*	− 0.58	− 0.39	1	0.17	0.79*	0.79*
Vanillic	− 0.32	− 0.08	0.79*	0.49	− 0.14	0.61	0.52	0.08	0.62	0.17	1	− 0.14	− 0.13
ABTS	0.90	0.98	− 0.17	0.44	0.76*	− 0.73*	0.58	− 0.90	− 0.61	0.79*	− 0.14	1	0.99
FRAP	0.91	0.99	− 0.08	0.40	0.73*	− 0.70	0.56	− 0.91	− 0.63	0.79*	− 0.13	0.99	1

*TFC* total flavonoid content, *TPC* total phenolic content, *ABTS* 2,2-azinobis-(3-ethylbenzthiazolin-6-sulfonic acid), *FRAP* ferric ion reducing antioxidant power.

*The observed correlation is significant at the p ≤ 0.05 level.

In comparison with the raw flours, the FSF and FCF had significantly (p ≤ 0.05) high TFC (4.12 and 14.16 mg QE/g), TPC (0.36 and 1.91 mg GAE/g) and antioxidant capacities (11.22% and 78.75% ABTS inhibition, 3.71 and 15.98 mM TE/g), respectively. Similarly, the FSP had increased levels of TPC (0.71 mg GAE/g) and FRAP (5.35 mM TE/g). The enhancement of the phenolic compounds and, consequently, the antioxidant capacity implies that the modifications by the activity of the probiotic culture, which resulted in a series of endogenous processes like the production of enzymes, enzymatic hydrolysis, the disintegration of cell walls and depolymerisation into phenolic monomers that led to greater concentrations of the free fractions of polyphenolics with potent antioxidant capacity^62,63^. Those modifications, in turn, brought about high differential concentrations of phenolic acids and flavonoids in the FCF [gallic acid (5630.93 µg/g), *p*-coumaric acid (1379.73 µg/g) and taxifolin (10,651.61 µg/g)], FSF [quercetin (121.51 µg/g)] and FSP [caffeic acid (16.10 µg/g), vanillic acid (43,000.99 µg/g), and luteolin (244.02 µg/g], respectively (Table 6).

Phenolic compounds are a group of bioactives that can contribute their phenoxyl hydrogen atom to neutralise free radical species and form phenoxyl radicals^64^. Thus, the inhibitory effect on the reactive oxygen species is beneficial to ameliorating cardiovascular risk factors^65^. Table 6 shows better concentrations of hydroxybenzoic acids (gallic and vanillic) and hydroxycinnamic acids (*p*-coumaric and caffeic) were noted for FCF and FSP, respectively. Likewise, Cheng et al.^66^ recorded increased concentrations of *p*-coumaric acid and total phenolic compounds expressed as gallic acid equivalents in fermented adzuki beans (*Vigna angularis*). Further to the maximum presence of the targeted flavonoids in the fermented flours [FCF (taxifolin) and FSP (luteolin)], FSF had improved quercetin content than other fermented flours. The observed flavonoids from different flavonoid subclasses have been reviewed to exhibit potential disease-combating activity^67^.

The germinated WGB flours showed significant reductions in the TFC (2.98 and 3.33 mg QE/g). A similar pattern was also noted for the TPC (0.83 mg GAE/g) and antioxidant capacity of the GCF. The observed decreases are consistent with prior reports on the germination of cowpea beans, pearl millet, and sorghum types for 48 h or 96 h^27,68,69^. The low concentrations of the total polyphenols and antioxidant capacity due to germination can be ascribed to the biological conversion to other phenolics, formation of complexes with available macromolecules or enhanced hydrolysis by enzymes, i.e., polyphenol oxidase^27,69^. In contrast, GSF had an increased TPC (0.48 mg GAE/g), which probably influenced the improved antioxidant capacities of the flour (9.75% ABTS inhibition, 4.78 mM TE/g). Endogenous enzyme activity, including the degradation of the cellular wall components, might have liberated the bound phenolics into new polyphenols^63^.

Notwithstanding, the targeted phenolics data (Table 6) revealed the high levels of some hydroxycinnamic acids [trans-ferulic (1340.56 µg/g) and sinapic (1448.32 µg/g)] in the GCF, which might be associated with the conversion of bound phenolics into free ones or microbial degradation of polymerised phenolic components. Likewise, Teixeira-Guedes et al.^70^ reported high ferulic acid in the extract from germinated cowpeas for 5 days. These bioactive acids have been documented to regulate and suppress reactive oxygen species-mediated pathological conditions^64,71^. More recently, improved lipid profiles and a consequent significant decrease in oxidised low-density lipoprotein-cholesterol, oxidative stress, and inflammatory biomarkers were the outcomes of administering a ferulic acid capsule (500 mg twice daily for 6 weeks) to hyperlipidemic patients^65^. Furthermore, ferulic acid (250 mg/kg) antioxidant and anti-inflammatory characteristics were reported to effectively stimulate a gastroprotective effect against indomethacin-induced gastric ulcers in rats^72^.

### ANFs in the raw and bioprocessed flours

Tannin and oxalate salt are commonly present ANFs in edible grains, roots and tuber and are documented to form complexes with nutrients and digestive enzymes^73,74^. Tannin and oxalate were the assayed ANFs in the raw and bioprocessed flours (Table 1). Varying significant (p ≤ 0.05) reductions in the tannin content were recorded for the fermented WGB flours (FCF: 6.02 and FSF: 5.84 mg TAE/g) and the FSP (16.42 mg TAE/g). The reduced tannins have been related to microbial hydrolysis by various synthesised enzymes like tannin acyl hydrolases^75^, and the trends corroborate with prior findings for fermented millet and Bambara groundnut condiment^36,75^. The GCF and GSF exhibited significantly (p ≤ 0.05) decreased levels of tannin (4.89 and 4.06 mg TAE/g, respectively). Similar tannin content (4.38 mg catechin equivalent/g dry basis) has been previously reported for white sorghum malt^69^. The much higher tannin reductions in the germinated flours can be traced to the tannin solubilisation in soaking water and subsequent polyphenol oxidase-induced oxidative degradation during germination^75,76^.

Oxalate is an undesirable ANF, owing to its ability to lessen calcium absorption while occurring as calcium oxalate in the blood and consequently encouraging the development of kidney stones^74^. Although the oxalate contents in the flours were generally low compared to tannin content, oxalate declined concentrations were equally noted for the fermented flours (FCF: 2.52, FSF: 0.44 and FSP: 2.21 mg/g). Comparably, Ojokoh et al.^77^ reported low and high oxalate contents for fermented cowpea (1.30 mg/g) and sorghum (1.04 mg/g) flours, respectively. Fermentation-induced oxalate reduction may be linked to enzymatic degradation of oxalate^36^. In the same vein, the possible leaching of oxalates in soaking water^78^ and further enzymatic activity during germination could have brought about a significant reduction in the oxalate content of GCF (1.73 mg/g).

Interestingly, the degradation of oxalate, an organic acid chelator, was consistent with the elevated concentrations of oxalic acid. In a related trend, the increased concentrations of oxalic acid significantly (p ≤ 0.05) correlated with trans-ferulic (R^2^ = 0.75) and *p*-coumaric (R^2^ = 0.80) acids (Table 4). This could mean that the degraded oxalates might have partly contributed to the unbinding of oxalic acid alongside some phenolic compounds like ferulic and *p*-coumaric acids.

### Proximate composition

The proximate composition of the raw, fermented, and germinated whole cowpea, sorghum, and OFSP flours is presented in Fig. 1. In terms of the ash content, there were significant increases (p ≤ 0.05) in the bioprocessed cowpea (3.60%) and FSP flours (3.60%). The probable dissociation of minerals from ANFs (oxalate and tannin, Table 1), enhanced solubility and bioavailability of minerals at higher acidity induced by microbial hydrolytic enzymes may be related to the observed ash increases^27,44^. On the other hand, the non-significant differences (p ≤ 0.05) in the ash content recorded for the fermented and germinated sorghum flours could suggest that the bioprocessing effects were substrate-based.

The GCF and GSF showed notable increases in crude fibre levels (5.04% and 2.17%, respectively). The crude fibre content in the GCF is significantly higher (p ≤ 0.05) than in the fermented flours [FCF (4.11%) and FSP (3.20%)]. The enhanced crude fibre in the germinated flours could be attributed to cellular structure formation during germination^16^. Also, it might strongly account for the dried radicles (containing certain fibres) as part of the flour constituents. Nonetheless, there were significant (p ≤ 0.05) increases in the crude fibre content of FCF and FSP indicated. Kewuyemi et al.^27^ reported a similar crude fibre increase in the spontaneously fermented cowpea and suggested the enhanced level was due to selective microbial actions on the fibre fractions during semi solid-state fermentation. Regarding the fat levels of the flours, non-significant differences (p ≤ 0.05) existed between the cowpea flours. Whereas FSF, GSF and FSP showed significantly (p ≤ 0.05) reduced fat levels. The decreased fat contents of the flours are presumably due to the metabolism of microorganisms, the use of lipids as a nourishment source for fermenting organisms, or the lipolytic enzyme activity that breaks down lipids^44,79^.

All the bioprocessed flours had significantly enhanced percentage levels of protein contents. While the increased enzymatic activities during fermentation might have disintegrated complex protein structures into amino acids^79^, the enzyme synthesis of new protein monomers during fermentation and germination could have also improved the amino acids^79,80^. In addition, the significant reduction in the examined ANFs (Table 1) might partly contribute to the increased protein contents of the bioprocessed flours. The FCF and FSF exhibited significantly (p ≤ 0.05) improved carbohydrate (64.03% and 80.42%, respectively) and energy values (354.22 and 395.15 kcal/g, respectively). The increase in the carbohydrate level of fermented flours has been related to the probable enzymatic conversion of resistant starches to available starches^79^. Augmented OAs during the fermentation of cowpea and sorghum might have promoted the release of energy from amino acids, lipids, and sugars, which can be ascribed to the enhanced energy in the derived fermented flours. Regarding the germinated flours, GCF showed a non-significant difference in the energy value, but GSF had significant increases in the carbohydrate (75.16%) and energy value (378.21 kcal/g), indicating a higher calorific value due to the accumulated soluble sugars during germintaion.

### In vitro starch digestibility and insoluble dietary fibres

Digestion of carbohydrates under acidic conditions can impair absorption as otherwise in alkaline conditions^9,81^. Lactic acid fermentation of the WGB revealed significant (p ≤ 0.05) reductions in the RDS (FCF: 19.94 g/100 g and FSF: 18.07 g/100 g) and subsequently increased levels of SDS (FCF: 2.53 g/100 g and FSF: 2.35 g/100 g) (Table 8). Primarily, the formation of lactic acid (FCF: 251.44 and FSF: 127.69 mg/kg, Table 3) and, consequently, the acid inhibitory impact on amylolysis might have contributed to the reduced starch digestion rate of the flours^55,82^. On the other hand, the improved concentration of propionic acid (FCF: 67.83 and FSF: 7.88 mg/kg, Table 3) linked to glycaemia may be assumed beneficial for delaying gastric emptying rate^55,83^. Contrarily, high RDS (19.26 g/100 g) and concomitant low SDS (0.59 g/100 g) were noted for the FSP. Akanbi et al.^84^ reported a high hydrolysis index (linked to starch digestibility) for the unmodified OFSP starch. Low extractable starch and high amylopectin proportions were previously reported for an OFSP cultivar^85^. The probable explanation for the increased RDS in FSP might thus be related to the starch content and amylopectin ratio (an easily accessible starch component for alpha-amylase attack)^84,86^.

**Table 8:** In vitro starch digestibility and insoluble dietary fibres in the raw, fermented, and germinated WGB and OFSP flours.

Samples	RDS (g/100 g)	SDS (g/100 g)	TDS (g/100 g)	RS (g/100 g)	ADF (%)	HMC (%)	NDF (%)
RCF	21.15^f^ [0.16]	1.26^b^ [0.20]	23.09^e^ [0.19]	42.19^b^ [0.16]	8.00^b^ [0]	2.94^b^ [0.10]	10.94^d^ [0.10]
FCF	19.94^de^ [0.04]	2.53^d^ [0.23]	21.22^d^ [0.19]	45.00^c^ [0.35]	8.08^b^ [0.08]	5.93^d^ [0.11]	14.01^f^ [0.19]
GCF	23.25^g^ [0.10]	0.54^a^ [0.13]	20.74^d^ [0.28]	41.43^b^ [1.21]	12.12^c^ [0.12]	5.59^d^ [0.10]	17.71^g^ [0.22]
RSF	20.36^e^ [0.41]	1.77^c^ [0.10]	19.77^c^ [0.25]	27.51^a^ [0.22]	4.08^a^ [0]	3.87^c^ [0.06]	7.95^b^ [0.06]
FSF	18.07^b^ [0.23]	2.35^d^ [0.18]	19.93^c^ [0.08]	56.73^d^ [0.09]	4.00^a^ [0]	1.53^a^ [0.40]	5.53^a^ [0.40]
GSF	19.68^cd^ [0.12]	1.74^c^ [0.02]	17.37^a^ [0.03]	64.43^e^ [0.25]	4.08^a^ [0]	8.23^f^ [0.31]	12.31^e^ [0.31]
RSP	16.73^a^ [0.01]	1.57^bc^ [0.10]	19.07^b^ [0.31]	42.38^b^ [0.14]	4.08^a^ [0]	6.08^d^ [0.09]	10.17^c^ [0.09]
FSP	19.26^c^ [0.16]	0.59^a^ [0.16]	19.50^bc^ [0.25]	46.28^c^ [0.38]	4.00^a^ [0]	6.70^e^ [0.06]	10.70^cd^ [0.06]

Values in square brackets are the standard deviations of the respective means with varying superscripts that are significantly different (p ≤ 0.05) per column.

*WGB* whole grains and beans, *OFSP* orange-fleshed sweet potato, *ADF* acid detergent fibre, *NDF* neutral detergent fibre, *HMC* hemicellulose, *RDS* rapidly digestible starch, *RS* resistant starch, *SDS* slowly digestible starch, *TDS* Total digestible starch, *RCF* raw cowpea flour, *RSF* raw sorghum flour, *RSP* raw orange-fleshed sweet potato flour, *FCF* fermented cowpea flour, *FSF* fermented sorghum flour, *FSP* fermented orange-fleshed sweet potato flour, *GCF* germinated cowpea flour, *GSF* germinated sorghum flour.

In alignment with the increase in digestibility recorded for germinated cowpeas by Benítez et al.^7^, GCF showed significantly (p ≤ 0.05) reduced SDS levels (0.54 g/100 g) with accompanying significantly (p ≤ 0.05) high RDS (23.25 g/100 g). The degradation of highly branched amylopectin and unbranched amylose into simple sugars under a potentially favourable pH (6.20, Table 1) might account for the observed high starch digestibility^44^. Also, the increased RDS could be related to the partial but significant degradation of tannins and oxalates in GCF (Table 1) that possibly enhanced the enzymatic susceptibility of its starch^7^. Nevertheless, non-significantly (p ≤ 0.05) different SDS levels were recorded between RSF (1.77 g/100 g) and GSF (1.74 g/100 g). Compared to the fermented flours, GCF (20.74 g/100 g) and GSF (17.37 g/100 g) consistently displayed greater total digestible starch reductions. The observation agreed with the reduced digestible starch contents recorded for germinated finger millet^80^ and pigeon pea flours^87^. It was linked with enhanced amylase activity during germination that could have resulted in starch degradation into mono- and oligosaccharides^80,87^.

The RS levels in the fermented flours were notably increased across the different substrates experimented. The highest RS content was recorded for FSF (56.73 g/100 g), followed by FSP (46.28 g/100 g) and FCF (45.00 g/100 g). Such RS increases have been previously reported for fermented potato flour^88^ and chemically modified sorghum starch^89^. The rise in RS content could be linked to the liberated metabolites during fermentation that likely led to the formation of a large starch chain (for example, esterification of the carboxyl group with the hydroxyl group of lactic acid on the starch chain) and provided steric restraint for digestive enzymes^88,89^. Particularly for FSF exhibiting more than one-fold RS increase, the plausible presence of enhanced components like amino acids, polyphenols and other substances that hinder starch digestion is attributable to the increased RS content^88^. The starch in materials has been reported to interact with sugars; thereby, it either restricts or extends the rate of hydrolysis depending on the starch source^13,90^. The unhydrolysed starch in RCF (42.19 g/100 g) and GCF (41.43 g/100 g) were insignificantly (p ≤ 0.05) different, and this probably conforms with the high RDS in GCF (23.25 g/100 g, Table 8). Such that the sugars liberated during the bean germination might have extended the rate of starch hydrolysis. In contrast, significantly (p ≤ 0.05) increased RS content in GSF (64.43 g/100 g) was recorded. The restricted hydrolysis in GSF (decreased RDS, 19.68 g/100 g), consequently due to the stimulation of the sugar type, can thus be attributed to the enhanced RS content in the GSF.

The ADF comprises mainly cellulose and lignin, while the NDF predominantly consists of cellulose, HMC, and lignin. As presented in Table 8, the cowpea samples generally had significantly (p ≤ 0.05) higher levels of cellulose and lignin (8.00–12.12%). The sorghum and OFSP samples showed insignificantly (p ≤ 0.05) different values and were within 4.00–4.08%. These variations are in accordance with the high quantity of the TDF reported for pulses (TDF 14–32%, soluble fibre 4–5%) as compared to whole grain cereals (TDF 6–22%, soluble fibre 0.5–1%) and OFSP (TDF 2.01–3.23%, soluble fibre 0.15–1%)^6,8^. There is scarce literature data that reports the specific insoluble fibre fractions of raw pulses, OFSP and their fermented or germinated forms^6,44^. Notwithstanding, the increase in the ADF and NDF (12.12% and 17.71%, respectively) of the GCF can be attributed to the presence of emerging radicles from the germinated beans that contain more cellulose and lignin (12.12%) as well as HMC (5.59%) as structural components. In a similar trend, the HMC level in the GSF (8.23%) contributed more to its NDF (12.31%).

Contrasting HMC contents were recorded for the fermented flours (Table 8). While the FCF (5.93%) and FSP (6.70%) showed significantly increased HMC levels, the FSF had reduced HMC content (1.53%). Vidal-Valverde et al.^91^ found increased lignin but reduced cellulose and HMC in naturally fermented lentils. The deviations in the insoluble fibre fractions could infer that the microbial activities during probiotic fermentation had selective effects on the HMC fraction in the FCF and FSP. The significant solubilisation of HMC in the FSF is due to the low pH (3.89) that partly inactivates amylases, particularly α-amylase, such that it lessens the enzyme actions to deactivate potential enzyme-degrading fibrous components^92^.

## Conclusion

The present study explored the same short-term germination and probiotic fermentation temperature and time and evaluated the consequent effect on the derived flours from whole cowpea, sorghum, and OFSP. The microbial and enzymatic modification extent of the dual bioprocessing approaches was found to be dependent on the food substrate type and composition. The FCF had the highest TTA, corresponding to high OAs, total flavonoid and phenolic contents, antioxidant capacity, and SDS. In contrast, the GCF exhibited mild acidity, better concentrations of some OAs (fumaric, l-ascorbic and oxalic acids), phenolic compounds (trans-ferulic and sinapic acids), the highest protein, crude fibre and insoluble dietary fibres. The FSF showed the least oxalate content and highest energy value, reduced RDS and enhanced SDS. On the other hand, the GSF revealed the lowest tannin and highest RS and HMC contents. Lastly, the FSP displayed the lowest fat, highest concentrations of some targeted phenolic compounds (caffeic acid, luteolin and vanillic acid) and comparably high ash content. Moreover, the mixture of the bioprocessed flours in reference to the unique features may result in diverse wellness-targeted finished foods. Further insight into the technological characteristics of the bioprocessed flours would broaden formulation choice.

## Data Availability

The data reported in this study are available within the article and may be made available from the corresponding author upon reasonable request.

## Abbreviations

ABTS: 2,2-Azinobis-(3-ethylbenzthiazolin-6-sulfonic acid)
ADF: Acid detergent fibre
ANFs: Antinutritional factors
FCF: Fermented cowpea flour
FRAP: Ferric ion reducing antioxidant power
FSF: Fermented sorghum flour
FSP: Fermented orange-fleshed sweet potato flour
GCF: Germinated cowpea flour
GSF: Germinated sorghum flour
HMC: Hemicellulose
LAB: Lactic acid bacteria
NDF: Neutral detergent fibre
OAs: Organic acids
OFSP: Orange-fleshed sweet potato
RCF: Raw cowpea flour
RDS: Rapidly digestible starch
RS: Resistant starch
RSF: Raw sorghum flour
RSP: Raw orange-fleshed sweet potato flour
SDS: Slowly digestible starch
TDF: Total dietary fibre
TFC: Total flavonoid content
TPC: Total phenolic content
TTA: Total titratable acidity
UHPLC-PDA: Ultra-high performance liquid chromatography-photo diode array
WGB: Whole grains and beans
